# Dual-tracer ^18^F-FDG and ^18^F-AlF-NOTA-octreotide PET/CT imaging in oncocytic thyroid adenoma: a case report and literature review

**DOI:** 10.3389/fendo.2025.1612851

**Published:** 2025-08-29

**Authors:** Jing Li

**Affiliations:** ^1^ Department of Nuclear Medicine, The Second Affiliated Hospital, Zhejiang University School of Medicine, Hangzhou, China; ^2^ Institute of Nuclear Medicine and Molecular Imaging of Zhejiang University, Hangzhou, China; ^3^ Key Laboratory of Medical Molecular Imaging of Zhejiang Province, Hangzhou, China

**Keywords:** ^18^F-fluorodeoxyglucose, ^18^F-AlF-NOTA-octreotide, PET/CT, oncocytic thyroid adenoma, somatostatin receptors

## Abstract

**Introduction:**

oncocytic thyroid adenoma (OA), a rare benign neoplasm, poses diagnostic challenges in conventional imaging modalities. The study investigates the diagnostic utility of dual-tracer ^18^F-fluorodeoxyglucose (^18^F-FDG) and ^18^F-AlF-NOTA-octreotide (^18^F-OC) positron emission tomography/computed tomography (PET/CT) in characterizing OA.

**Methods:**

We present a histopathologically confirmed OA case evaluated through simultaneous ^18^F-FDG and ^18^F-OC PET/CT imaging. Quantitative analysis included maximum standardized uptake value (SUVmax), mean standardized uptake value (SUVmean), and metabolic tumor volume (MTV). A systematic literature review was conducted to contextualize the imaging findings within existing evidence.

**Results:**

A 44-year-old female presenting with ACTH-independent Cushing syndrome (morning cortisol 632.8 nmol/L, non-suppressed on dexamethasone suppression tests) underwent comprehensive endocrine evaluation. Incidentally detected thyroid nodule demonstrated discordant tracer uptake: ^18^F-FDG PET/CT revealed intense hypermetabolism (SUVmax=31.68; SUVmean=9.18; MTV=4.33 cm^3^), while ^18^F-OC PET/CT showed moderate uptake (SUVmax=6.86; SUVmean=4.41; MTV=1.72 cm^3^). Histopathology confirmed OA with negative SSTR2 expression. Postoperative cortisol levels remained elevated. After nearly two years of clinical follow-up with conservative management using symptomatic medication, she underwent pituitary lesion resection, which confirmed a pituitary adenoma/pituitary neuroendocrine tumor (PitNET) consistent with a densely granulated corticotroph tumor.

**Conclusion:**

This case demonstrates the complementary diagnostic value of dual-tracer PET/CT in OA characterization, particularly in SSTR2-negative variants. The discordant uptake patterns suggest distinct metabolic pathways in OA pathophysiology. Prospective multicenter studies with larger cohorts are warranted to establish standardized diagnostic criteria and explore theranostic applications of these imaging biomarkers in OA.

## Introduction

Thyroid oncocytic tumors, originating from follicular cells, are defined by the presence of ≥75% oncocytes. The 2022 World Health Organization (WHO) Classification of Thyroid Neoplasms stratifies these lesions into benign oncocytic thyroid adenomas (OA) and malignant oncocytic thyroid carcinoma (OCA) (1, 2). While ultrasonography remains the primary imaging modality for thyroid nodule evaluation, its diagnostic specificity for OA remains limited due to overlapping radiological features with other thyroid neoplasms (3). Recent advancements in molecular imaging have positioned positron emission tomography/computed tomography (PET/CT) as a pivotal tool for tumor characterization, particularly through ^18^F-fluorodeoxyglucose (^18^F-FDG) imaging which demonstrates increasing utilization in thyroid tumor detection (4). Notably, ^18^F-AlF-NOTA-octreotide (^18^F-OC), a novel somatostatin receptor (SSTR)-targeted PET tracer, has shown diagnostic potential in neuroendocrine tumors (5), yet its application in thyroid oncocytic pathology remains underexplored. This study pioneers the dual-tracer ^18^F-FDG/^18^F-OC PET/CT evaluation of a histologically confirmed OA case, coupled with systematic literature synthesis, to elucidate the imaging signatures and clinical implications of this multimodal approach.

## Methods

This study data from a 44-year-old female patient suspected of having Cushing’s syndrome. In September 2022, to localize the source of ACTH overproduction, the patient underwent ^18^F-FDG and ^18^F-OC PET/CT imaging, which revealed a thyroid lesion with abnormal uptake. The lesion was later confirmed as OA through biopsy and surgical resection pathology. The clinical manifestations and pathological features were analyzed. This study was approved by the Ethics Committee of the Second Affiliated Hospital of Zhejiang University School of Medicine (No. 2025-0574).

The PET/CT imaging was performed using a Siemens Biograph mCTx PET/CT scanner from Germany. Both ^18^F-FDG and ^18^F-OC were prepared in-house using a CYRIS HM-12 cyclotron from Sumitomo Heavy Industries, Japan, and an AllinOne multifunctional synthesis module from Beijing PET Technology Co., Ltd.

The patient fasted for ≥6 hours, with a blood glucose level of 5.4 mmol/L. An intravenous injection of ^18^F-FDG was administered with an activity of 444.0 MBq. After the injection, the patient rested quietly for 71 minutes, emptied the bladder, and drank water to fill the stomach wall before undergoing ^18^F-FDG PET/CT imaging. The imaging range extended from the top of the skull to the mid-upper thigh. A CT body scan was performed first,with a tube voltage of 120 kV, using the CARE-Dose4D technology to automatically control the tube current, and a slice thickness of 5.0 mm. This was followed by a PET body scan, with 1 minute per position, 6 bed positions. The TrueX+TOF post-processing algorithm was used for image reconstruction, yielding PET images, CT images, and PET/CT fusion images. After an interval of one day, the patient did not need to fast. An intravenous injection of ^18^F-OC was administered with an activity of 425.5 MBq. After the injection, the patient rested quietly for 71 minutes, emptied the bladder, and then underwent ^18^F-OC PET/CT imaging. The imaging protocol was the same as that for ^18^F-FDG PET/CT.

The PET/CT images from both scans were imported into the Siemens syngo.via software on the imaging workstation. The region of interest (ROI) for the thyroid lesion was manually delineated, and the software automatically calculated the maximum standardized uptake value (SUVmax), average standardized uptake value (SUVmean), and metabolic tumor volume (MTV) of the lesion. In this study, MTV was defined as the volume above SUVmax = 2.5 threshold. Additionally, the SUVmax and SUVmean of the contralateral thyroid tissue were measured.

## Results

### Case report

A 44-year-old female was suspected of having Cushing’s syndrome based on elevated cortisol and adrenocorticotropic hormone (ACTH) levels, with both standard and low-dose dexamethasone suppression tests showing non-suppression. Physical examination revealed moon facies, buffalo hump, central obesity, facial plethora, and abdominal striae. There was no history of familial or neoplastic diseases. Laboratory investigation revealed cortisol (8:00 AM - 4:00 PM - 0:00 AM) at 632.8- 310.9- 186.6 nmol/L, 24-hour urinary free cortisol at 93.0 μg/24h (normal range, 3.5-45.0μg/24h), adrenocorticotropic hormone (ACTH) (8:00 AM - 4:00 PM - 0:00 AM) at 48.4 - 35.8 - 21.2 pg/ml, Insulin-like growth factor-binding protein-3 (IGFBP-3) at 1.92 μg/mL (normal range, 3.3-6.6 μg/mL), total triiodothyronine (TT3) at 0.91 nmol/L (normal range, 0.98-2.33 nmol/L), thyroid-stimulating hormone (TSH) at 0.2 mIU/L (normal range, 0.35-4.94 mIU/L), thyroglobulin (TG) at 89.44 μg/L (normal range, 3.5-77.0 μg/L), CEA at 7.2 ng/mL (normal range, < 5 ng/mL), CA125 at 39.9 U/mL (normal range, < 35 U/mL).

**Figure 1:**
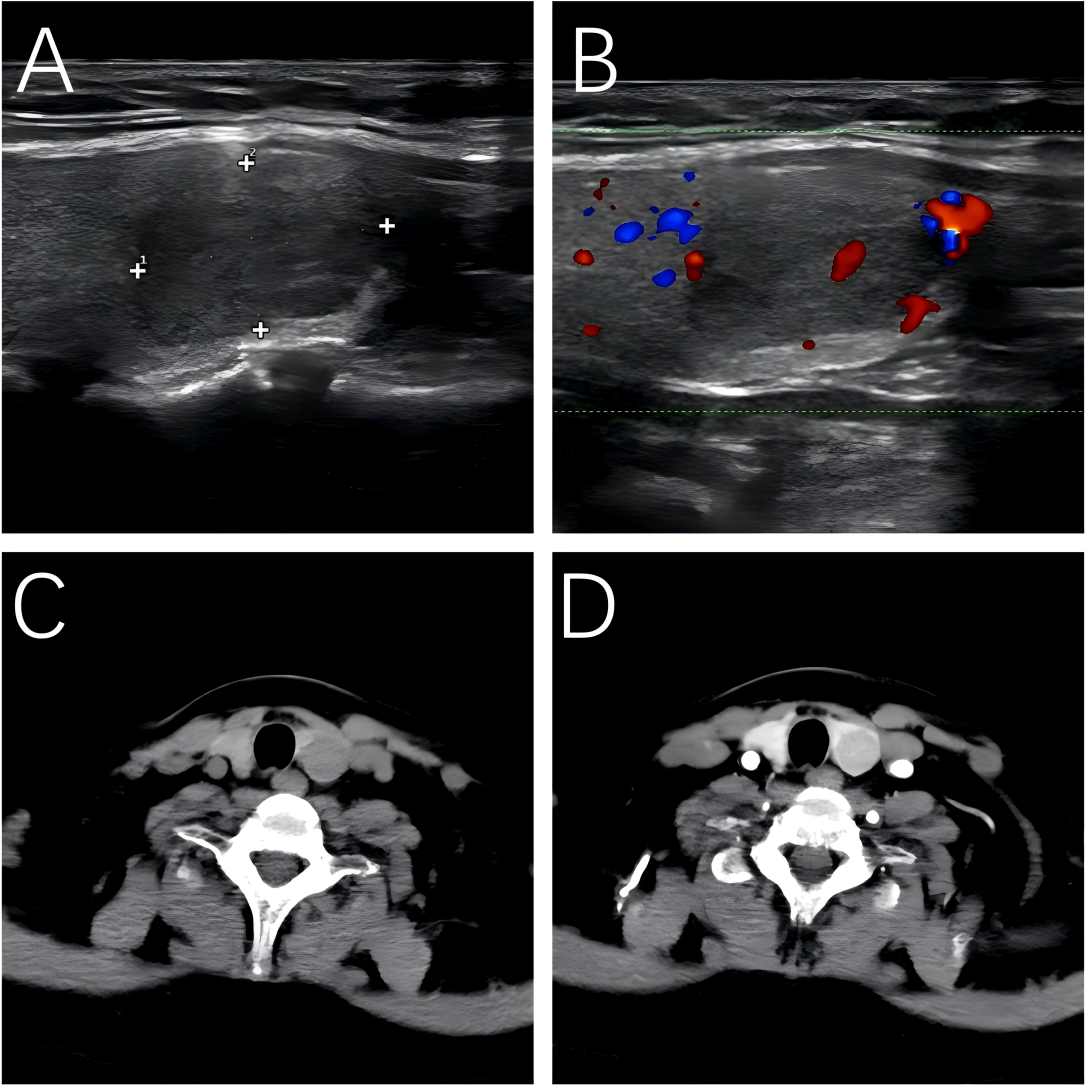
Ultrasound and CT-enhanced imaging of OA. **(A)** Ultrasound. **(B)** Color Doppler ultrasonography. **(C)** Axial view in CT scan. **(D)** Axial view in CT-enhanced scan.

Conventional imaging revealed a slightly hypointense lesion in the posterior pituitary on contrast-enhanced magnetic resonance imaging (MRI), with Rathke’s cleft cyst not excluded. Both adrenal ultrasonography and contrast-enhanced CT showed no significant abnormalities. Ultrasonography revealed a hypoechoic nodule measuring 1.65*1.09 cm in the lower pole of the left thyroid lobe. The nodule had well-defined margins, heterogeneous internal echogenicity, and a hypoechoic halo around it. Color Doppler imaging showed punctate blood flow signals within the nodule, and it was classified as TI-RADS category 3. Contrast-enhanced CT scan of the thyroid showed slight enlargement of the left thyroid lobe, with a hypodense nodule measuring approximately 1.6*1.5*1.7 cm in the mid-to-lower pole. The nodule exhibited slightly less enhancement compared to the surrounding thyroid parenchyma, with multiple punctate areas of significant enhancement within the nodule. The margins were clear and smooth. These findings suggested a benign nodule in the left thyroid lobe (**Figure 1**).

Subsequent ^18^F-FDG PET/CT revealed a hypodense nodule in the left thyroid lobe, measuring approximately 1.80*1.50 cm. The nodule exhibited significantly high metabolic uptake (SUVmean = 9.18, SUVmax = 31.68, MTV = 4.33 cm^3^), which was markedly higher than that of the contralateral thyroid tissue (SUVmean = 1.31, SUVmax = 1.77). No abnormal ^18^F-FDG uptake was demonstrated in the pituitary gland (SUVmean = 3.61, SUVmax = 6.83, MTV = 1.06 cm^3^). One day later, ^18^F-OC PET/CT also demonstrated high metabolic uptake in the same thyroid nodule (SUVmean = 4.41, SUVmax = 6.86, MTV = 1.72 cm^3^), which was higher than that of the contralateral thyroid tissue (SUVmean = 1.35, SUVmax = 2.08). No abnormal ^18^F-OC uptake was demonstrated in the pituitary gland (SUVmean = 3.69, SUVmax = 6.76, MTV = 0.55 cm^3^). Multidisciplinary team (MDT) discussion concluded that ectopic ACTH syndrome originating from the thyroid was highly likely.

**Figure 2:**
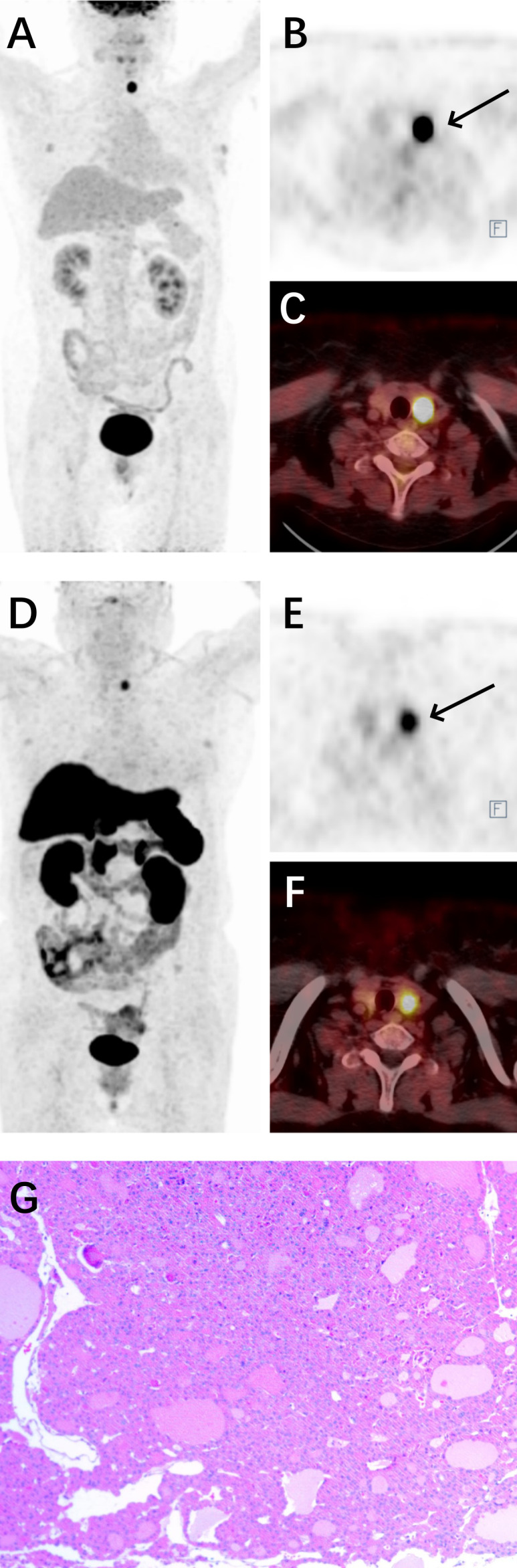
^18^F-FDG combined with ^18^F-OC PET/CT of OA and surgical pathology. **(A)** Maximum-intensity projection (MIP) image of the patient in ^18^F-FDG PET. **(B)** Axial view of the tumor in ^18^F-FDG PET (black arrow). **(C)** Fusion image in ^18^F-FDG PET/CT. **(D)** MIP image of the patient in ^18^F-OC PET. **(E)** Axial view of the tumor in ^18^F-OC PET (black arrow). **(F)** Fusion image in ^18^F-OC PET/CT. **(G)** Surgical pathology of OA.

Ultrasound-guided fine-needle aspiration (FNA) biopsy of the thyroid nodule indicated an oncocytic tumor. One month later, the patient underwent left thyroidectomy and isthmusectomy, during which two lesions were identified in the left thyroid and isthmus, measuring 1.5*1.0 cm and 1.5*1.5 cm, respectively. The histopathological and immunohistochemical findings were consistent with a diagnosis of oncocytic adenoma. Immunohistochemical analysis revealed positive staining for TTF-1 (SPT24), PAX-8, TG, and CD56, and negative for Syn, CgA, INSM1, SF-1, T-PIT, PIT-1, GATA-3, ACTH, Prolactin, SSTR2, CEA (Mono), and P53. The proliferation index Ki-67 was 2%.(**Figure 2**)

Postoperative reevaluation revealed persistently elevated cortisol levels (8:00 AM - 4:00 PM - 0:00 AM: 235.7 - 225.9 - 221.3 nmol/L), indicating clinical non-remission. Follow-up MRI demonstrated a contrast-enhancing hypointense lesion in the left pituitary, measuring 3.8 × 2.4 mm, suggestive of a microadenoma with Rathke’s cleft cyst. To further localize the source, bilateral inferior petrosal sinus sampling (IPSS) was performed, showing central-to-peripheral ACTH gradient dominance, consistent with possible Cushing’s disease. MDT discussion concluded there was an indication for pituitary surgical exploration; however, the patient opted for conservative management with levothyroxine and ketoconazole.

Two years later, the patient underwent pituitary lesion resection. Histopathological examination confirmed a pituitary adenoma/PitNET, consistent with a densely granulated corticotroph tumor. Immunohistochemical analysis was positive for T-PIT, ACTH, CAM5.2, and Synaptophysin, but negative for PIT-1, SF-1,GATA-3, ER alpha, GH, Prolactin, TSH beta, LH beta, FSH beta, Alpha-Subunit, P53, and SSTR2. The proliferative index Ki-67 < 3%. Postoperative evaluation following pituitary surgery showed significantly decreased hormone levels.

## Discussion

The term “Hürthle cell” was first introduced by Karl Hürthle in 1894 when he characterized the parafollicular C cells of the canine thyroid gland in his histological studies (6). The evolving classification of oncocytic thyroid neoplasms reflects advancing molecular understanding: the 4th edition WHO classification (2017) reclassified Hürthle cell adenoma/carcinoma as distinct from follicular neoplasms (7), while the 5th edition (2022) adopted the histogenetically accurate terminology “oncocytic thyroid adenoma/carcinoma” (OA/OCA) to replace the eponymous designation (2).

OA and OCA represent relatively uncommon subtypes of thyroid neoplasms, demonstrating a statistically significant female predominance in incidence (8, 9). Notably, OCA shows distinct epidemiological characteristics with higher prevalence among elderly male patients and an increased propensity for lymph node metastasis compared to other thyroid tumor types (10, 11). FNA is the gold standard for evaluating thyroid tumors, offering high sensitivity and specificity (12, 13). However, distinguishing OA from OCA cannot rely solely on FNA; it requires surgical pathology to assess morphological features such as capsular and/or vascular invasion, infiltration into surrounding thyroid tissue, or extension beyond the thyroid. According to a multicenter study across six Asian countries (14), oncocytic-predominant nodules accounted for 1.8% (760/42,190) of all FNA samples, with OAs representing 28.5% (82/288) of all surgically followed-up cases.

OA is a rare benign tumor with well-differentiated histology, typically small in size, and characterized by follicular growth patterns. It lacks malignant features such as capsular or vascular invasion, extrathyroidal extension, or lymph node and systemic metastases. However, oncocytic tumors are more aggressive than follicular tumors, and there have been reports of malignant transformation and metastasis in cases of OA (8, 15). At the genetic level, OCA exhibits a more complex gene expression profile and chromosomal alterations. Studies have shown (16) that copy number alteration (CNA) patterns, particularly the genome haploidization type (GH-type), are predominantly observed in OCA, while OA shows milder changes. Compared to OCA, OA patients are typically younger, often by about 10 years (8). The clinical presentation of OA is often nonspecific and indistinguishable from other follicular thyroid tumors. Most patients are diagnosed incidentally during physical examinations, presenting with a painless, slowly growing solitary thyroid nodule or cervical lymphadenopathy.

Ultrasound is a commonly used imaging modality for thyroid tumors. The sonographic features of OA are diverse, often showing solid nodules, hypoechogenicity, well-defined margins, and abundant intranodular and perinodular blood flow. Cervical lymphadenopathy is generally absent, and no specific ultrasound feature is unique to OA (3). The size and ultrasound characteristics of the thyroid lesion in this case are consistent with previous literature (17). On CT imaging, the lesion appeared as a hypodense mass with well-defined margins, showing slightly less enhancement compared to the surrounding thyroid parenchyma after contrast administration, which aligns with findings reported in the literature (18).

^18^F-FDG, a glucose analogue, represents the most extensively utilized tracer in PET imaging for oncological diagnosis and therapeutic monitoring (4). It demonstrates abnormal uptake in malignant cells, yet elevated glucose metabolism may also occur in certain inflammatory lesions. The use of PET/CT for diagnosing thyroid oncocytic tumors remains controversial (19, 20). Under normal conditions, ^18^F-FDG uptake in thyroid cells is uniform and low-level, making it indistinguishable on PET/CT imaging. This phenomenon occurs because healthy thyroid cells preferentially utilize fatty acids as their primary metabolic substrate, while low-grade malignant tumor cells similarly do not rely predominantly on glucose metabolism. Therefore, PET/CT is not routinely recommended for diagnosing thyroid tumors. In this case, the OA exhibited significantly elevated ^18^F-FDG uptake, with an SUVmean of 9.18, an SUVmax of 31.68 and an MTV of 4.33 cm^3^. SUVmean, SUVmax, and MTV are common metabolic parameters in PET imaging, playing a crucial role in the diagnosis and treatment evaluation of tumors (21). Compared to other thyroid tumor subtypes, both OA and OCA consistently demonstrate significantly higher ^18^F-FDG uptake. Some researchers consequently recommend incorporating ^18^F-FDG PET imaging into the standard diagnostic workup for all suspected OA/OCA cases (22, 23). This is attributed to the abundant eosinophilic cytoplasm of oncocytic cells due to mitochondrial accumulation. These cells exhibit significantly higher uptake of ^18^F-FDG, compared to normal cells (24, 25).

Somatostatin (SST) is a naturally occurring peptide hormone containing 14 or 28 amino acids, widely distributed in the central nervous system and various peripheral tissues. It inhibits secretory processes in the pituitary, pancreas, and gastrointestinal tissues by binding to specific receptors on cell membranes (26). Octreotide, a synthetic SST analog containing 8 amino acids, shares similar properties with SST and binds with high affinity and specificity to somatostatin receptors (SSTR). Compared to normal tissues, SSTRs are often overexpressed in tumor cells of gastrointestinal pancreatic tumors, meningiomas, pituitary adenomas, carcinoids, small cell lung cancers and phosphaturic mesenchymal tumor (PMT) (27–29). In 2012, Laverman et al. reported that ^18^F-OC by conjugating octreotide with Al^18^F-labeled chelators. This tracer offers advantages such as high yield, moderate half-life, and suitability for centralized production and distribution, making it a widely recognized somatostatin analog tracer (30). Nonetheless, the use of ^18^F-OC PET/CT for the diagnosis and treatment of OA/OCA remains limited by the small number of cases studied. In this case of OA, the ^18^F-OC uptake was abnormally elevated, with an SUVmean of 4.41, an SUVmax of 6.86 and an MTV of 1.73 cm^3^. This suggests that OA may also exhibit overexpression of SSTR. Both SSTR and thyroid-stimulating hormone receptor (TSHR) are considered targets for the diagnosis and treatment of other tumors. GILLIS et al. (31) reported a significant negative correlation between somatostatin receptor type 2 (SSTR2) and TSHR expression in OCA patients, with increased SSTR2 positivity observed in advanced OCA but not in OA. Among 15 OA patients, only 2 showed SSTR2 positivity, while 11 exhibited TSHR positivity. In this case of OA, the patient’s TSH level was below the normal range, and SSTR2 was negative. However, the ^18^F-OC PET imaging still revealed abnormally high metabolic uptake. This suggests that ^18^F-OC PET imaging retains diagnostic value for OAs even in the absence of SSTR2 expression. The discordance between SSTR2 negativity and elevated ^18^F-OC uptake may suggest the involvement of alternative SSTR subtypes (e.g., SSTR5). Beyond ^18^F-OC, Tang et al. (32) reported abnormally high uptake of ^18^F-PSMA-1007 in OA. Aziz et al. (33) reported abnormally high uptake of ^18^F-fluorocholine in OA. The patient was ultimately diagnosed with pituitary-dependent Cushing’s disease. However, the pituitary lesion show no abnormal uptake on either ^18^F-FDG or ^18^F-OC PET/CT imaging. This lack of detection is likely attributable to the small size of the pituitary lesion, with MRI revealing a maximum diameter of only 3.8 mm. Additionally, normal pituitary cells express a certain level of SSTR. On ^18^F-OC PET imaging, this typically manifests as mild to moderate physiological uptake, which can also contribute to the underdetection of pituitary tumors (34).

## Conclusion

Dual-tracer ^18^F-FDG and ^18^F-OC PET/CT imaging have diagnostic value for OA. The combination of these two tracers in PET imaging can provide more comprehensive metabolic information about the tumor. However, the results must be integrated with clinical presentation, other imaging modalities, and pathological findings for a comprehensive analysis to improve diagnostic accuracy. Prospective multicenter studies with large cohorts are needed to further clarify the diagnostic and therapeutic significance of the dual-tracer PET/CT in OA.
